# Efficient cytometry analysis with FlowSOM in Python boosts interoperability with other single-cell tools

**DOI:** 10.1093/bioinformatics/btae179

**Published:** 2024-04-17

**Authors:** Artuur Couckuyt, Benjamin Rombaut, Yvan Saeys, Sofie Van Gassen

**Affiliations:** Department of Applied Mathematics, Computer Science and Statistics, Ghent University, 9000 Ghent, Belgium; Data Mining and Modelling for Biomedicine, VIB Center for Inflammation Research, 9052 Ghent, Belgium; Department of Applied Mathematics, Computer Science and Statistics, Ghent University, 9000 Ghent, Belgium; Data Mining and Modelling for Biomedicine, VIB Center for Inflammation Research, 9052 Ghent, Belgium; Department of Applied Mathematics, Computer Science and Statistics, Ghent University, 9000 Ghent, Belgium; Data Mining and Modelling for Biomedicine, VIB Center for Inflammation Research, 9052 Ghent, Belgium; Department of Applied Mathematics, Computer Science and Statistics, Ghent University, 9000 Ghent, Belgium; Data Mining and Modelling for Biomedicine, VIB Center for Inflammation Research, 9052 Ghent, Belgium

## Abstract

**Motivation:**

We describe a new Python implementation of FlowSOM, a clustering method for cytometry data.

**Results:**

This implementation is faster than the original version in R, better adapted to work with single-cell omics data including integration with current single-cell data structures and includes all the original visualizations, such as the star and pie plot.

**Availability and implementation:**

The FlowSOM Python implementation is freely available on GitHub: https://github.com/saeyslab/FlowSOM_Python.

## 1 Introduction

FlowSOM (Van Gassen *et al.* 2015, Quintelier *et al.* 2021) is an automated gating technique that is currently the standard in the cytometry field (Weber and Robinson 2016, Liu *et al.* 2019, Mathew *et al.* 2020, Gaebler *et al.* 2021). The algorithm consists of two main steps. Firstly, a self-organizing map (Kohonen 2001) is used as a low-level clustering and secondly, the cluster centers are subjected to a second consensus hierarchical clustering (referred to as metaclustering). These metaclusters typically correspond with cell types.

Currently, FlowSOM is implemented in R, a programming language renowned for its excellent visualization packages and statistical capabilities. Furthermore, it is popular for computational cytometry analysis. In this paper, we introduce FlowSOM in Python in order to reach a broader range of researchers and to make use of its many advantages. Python is increasingly used in the single-cell omics field due to its better scalability for large datasets, as well as the better support for scalable ML techniques. Moreover, it has lower running times (Fourment and Gillings 2008, Nagpal and Gabrani 2019) and its accessibility to packages such as Scanpy (Wolf *et al.* 2018), the scverse (Virshup *et al.* 2021), scikit-learn and Tensorflow make it more appealing for big data analysis.

Existing packages providing FlowSOM in Python suffer from limitations such as inadequate visualizations or the absence of consensus metaclustering when compared to R (Vettigli 2018, Hatchin 2023, pyFlowSOM 2023). Consequently, we have taken the initiative to develop a FlowSOM Python package that is not only fast and scalable but also incorporates the visualizations that are provided in the original R package.

## 2 Materials and methods

The Python implementation of FlowSOM follows the same steps outlined in R: (i) reading the FCS file, (ii) building the SOM, (iii) building the minimum spanning tree (MST), and (iv) metaclustering. The FCS file is loaded into an anndata object using the Pytometry module (Virshup *et al.* 2021, Buttner *et al.* 2022). The self-organizing map is implemented directly in Python, translated from the C code of the FlowSOM R package, accelerated with Numba just-in-time compilation (Lam *et al.* 2015). Afterward, an MST is constructed using igraph and a Kamada-Kawai (Kamada and Kawai 1989) layout is calculated. The SOM cluster centers are subsequently clustered using hierarchical consensus clustering (Monti 2003) with scikit-learn.

The clustering outcomes are stored in a mudata object which supports cross-language HDF5 functionality and consists of two distinct anndata objects representing two different modalities: the cell data and the cluster data (Bredikhin *et al.* 2022). Anndata, in itself, offers sparse data support, lazy operations, a PyTorch interface and is intrinsically connected to the scverse, which provides a broad ecosystem of single-cell omic tools. This interoperability extends toward CITE-seq data, where FlowSOM could be used to cluster the proteomic data and seamlessly integrate with tools such as TotalVI (Gayoso *et al.* 2021). On top of that, SOM clustering has also been used in spatial proteomics to phenotype highly multiplexed tissue imaging data (Liu *et al.* 2023), which underscores the many use cases of a Python implementation of FlowSOM.

The cell data modality, on the one hand, contains data on the cell level, with not only the intensity levels with corresponding marker and channel names but also clustering and metaclustering labels per cell. On the other hand, the cluster data contains data on the cluster level, such as the Kamada-Kawai layout, the metacluster label per cluster and percentages. All of this information is stored as observations (matrices) or variables within the mudata object. All function and variable names are adapted to the PEP 8 Python style guides. Future work includes implementing the remainder of the visualizations as in R, further broadening the documentation and implementing batch SOM to further speed up the algorithm.

## 3 Results

### 3.1 Visualizations

**Figure 1.**
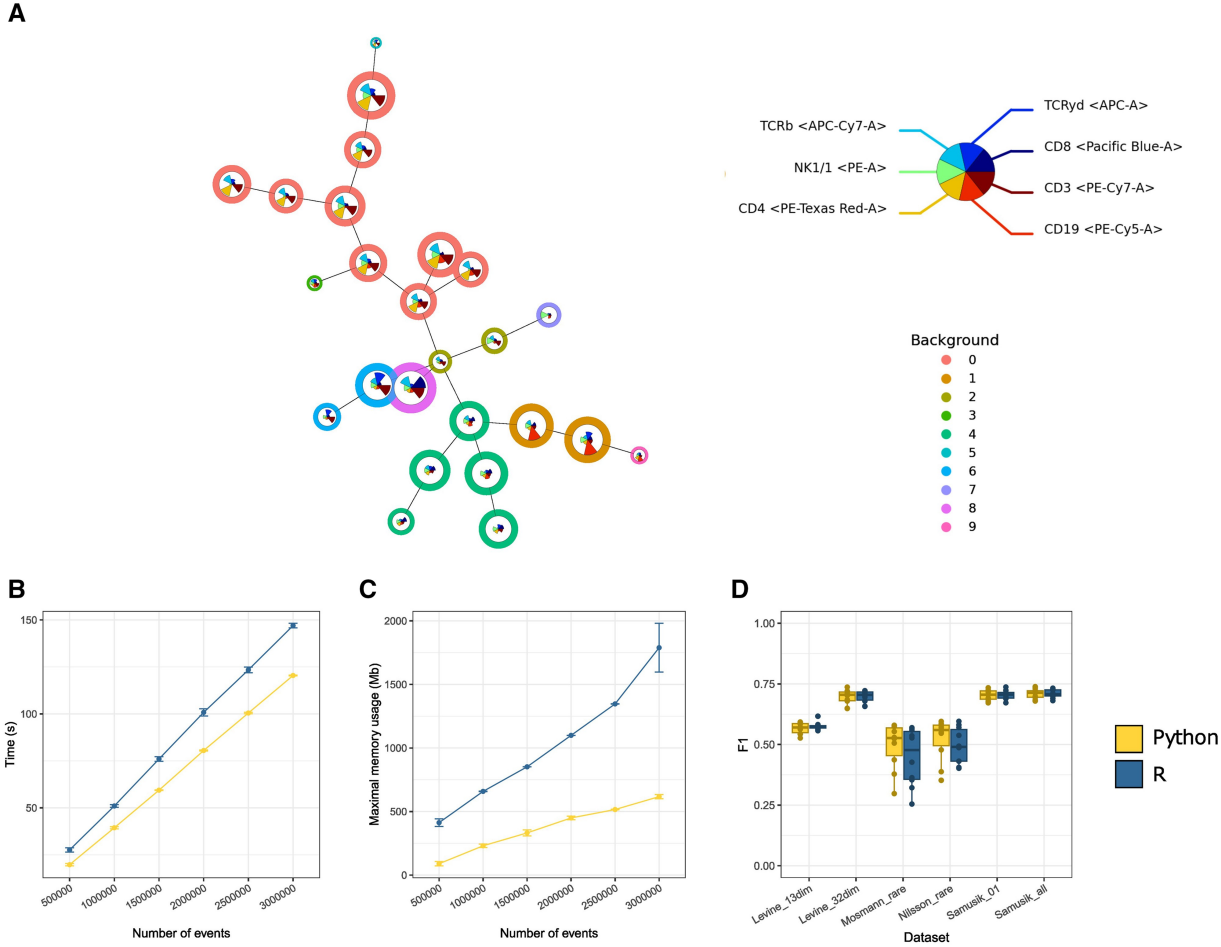
Overview of FlowSOM functionalities in Python. (A) A star plot of a FlowSOM object made in matplotlib. Each node is a cluster and is scaled according to the percentage of cells in this cluster. In each cluster, the marker median values are represented by the star charts. The metaclusters are displayed as the background color of each node. (B) The computational runtime of the Python implementation is lower than in R. The average computational runtime was tested on a single core of a compute cluster with 32 GB of RAM and we oversampled one file to the necessary amount of events. The error bars are the standard deviation over five runs. (C) FlowSOM in Python is more memory efficient than R. The memory usage was averaged over five runs on a single core of a compute cluster with 32 GB of RAM. We oversampled one file to the necessary amount of events. The error bars are the standard deviation over five runs. (D) The performance of the Python implementation of FlowSOM is equal to R. F1 scores are calculated based on the manually assigned cell labels and the values predicted from FlowSOM in both Python and R and are shown over 10 runs. Using a Wilcoxon rank-sum test, no significant differences were found (*P* > .05).

To recreate the star charts (Fig. 1A) and other important plots to visualize the FlowSOM objects in Python we made use of matplotlib. The clustering patterns and overall visual appearance exhibited remarkable consistency between the two implementations. A small remark is that the orientation of the stars varied slightly between Python and R (Supplementary Fig. S1).

### 3.2 Computational runtime and memory usage

To compare the runtime and memory usage, we made use of a single core on a compute cluster with 32 GB RAM. We used FlowSOM on the FlowSOM R demo FCS file with 7 colors (Van Gassen *et al.* 2015—https://github.com/saeyslab/FlowSOM), oversampled from 500 000 to 3 000 000 events with increments of 500 000 events and averaged the score over five runs.

We noticed that the Python version of FlowSOM outperforms its R counterpart in terms of execution speed (Fig. 1B). This difference became more pronounced as we scaled up the number of events. Specifically, we noted on average an 8-second difference when analyzing a dataset with 500 000 events and a 27-second difference when dealing with a dataset of 3 000 000 events. The execution speed did not change significantly when running the algorithm on different number of threads using the NUMBA_NUM_THREADS environment variable.

In our memory usage comparison (Fig. 1C), Python consistently demonstrates better memory efficiency compared to R. Similar to the computational runtime, the difference is more noticeable as the number of events increases. On average, when clustering an FCS with 500 000 events, Python consumes 323 Mb less memory than R. This gap increases, with Python using 1172 Mb less memory when handling an FCS containing 3 000 000 events.

### 3.3 Performance

To evaluate the algorithm's performance, we ran FlowSOM on six datasets, as detailed in Weber *et al.* (Weber and Robinson 2016). These datasets are Levine_13dim, Levine_32dim, Mosmann_rare, Nilsson_rare, Samusik_01 and Samusik_all. Following the analysis, we compared the F1-score and the purity of the clusters and metaclusters obtained from five iterations of both the Python and R implementations and compared them with the manually assigned cell labels (Fig. 1D, Supplementary Fig. S2). Despite the inherent stochastic variability and minor code differences in the metaclustering algorithm, we did not observe any significant differences in either the F1 score or the purity between the two implementations.

### 3.4 Conclusion

We have implemented FlowSOM, a popular clustering package, in Python to meet the growing demand for Python-based solutions in the field of computational cytometry. From there, it will improve the interoperability with existing single-cell omics tools with the help of anndata. This implementation meets the metaclustering performance set by the R package and surpasses them in terms of computational runtime and memory usage.

## Supplementary Material

btae179_Supplementary_Data

## Data Availability

The code and main example data is available at https://github.com/saeyslab/FlowSOM_Python. The data used for accuracy testing is available from http://flowrepository.org/id/FR-FCM-ZZPH.
