# Supplementary material for: Efficient cytometry analysis with FlowSOM in Python boosts interoperability with other single-cell tools
Source: Bioinformatics. 2024 Apr 17;40(4):btae179. doi: 10.1093/bioinformatics/btae179 (PMC11052654; doi:10.1093/bioinformatics/btae179)
Supplement: btae179_Supplementary_Data [file btae179_supplementary_data.docx]

Supplementary


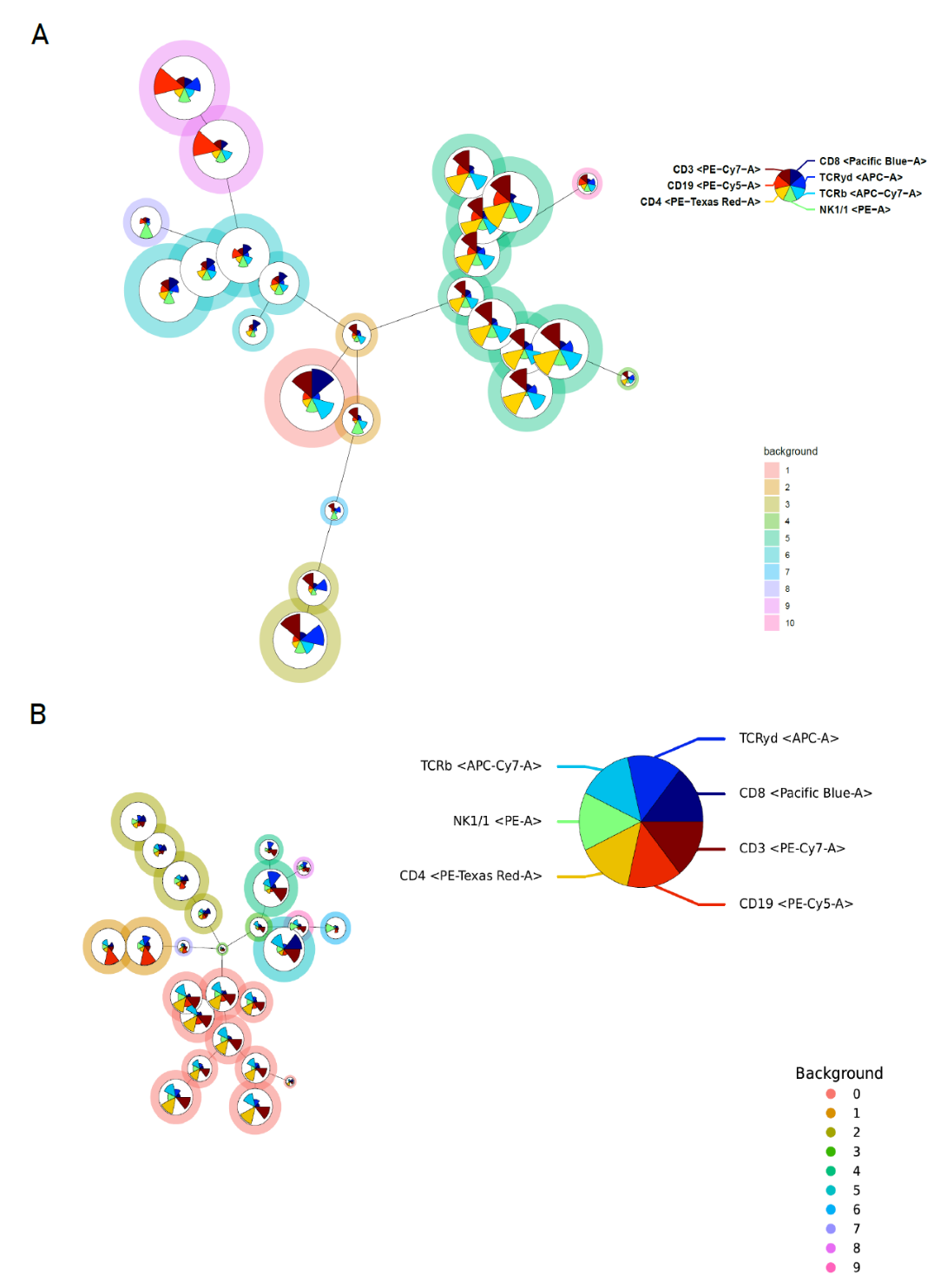


**Figure S1: Visual comparison of the PlotStars function in R versus the plot_stars function in Python. A:** Visualisation in R where the stars charts start from a vertical position and go clockwise whereas in **B** in Python the stars start horizontally and go counterclockwise.

**
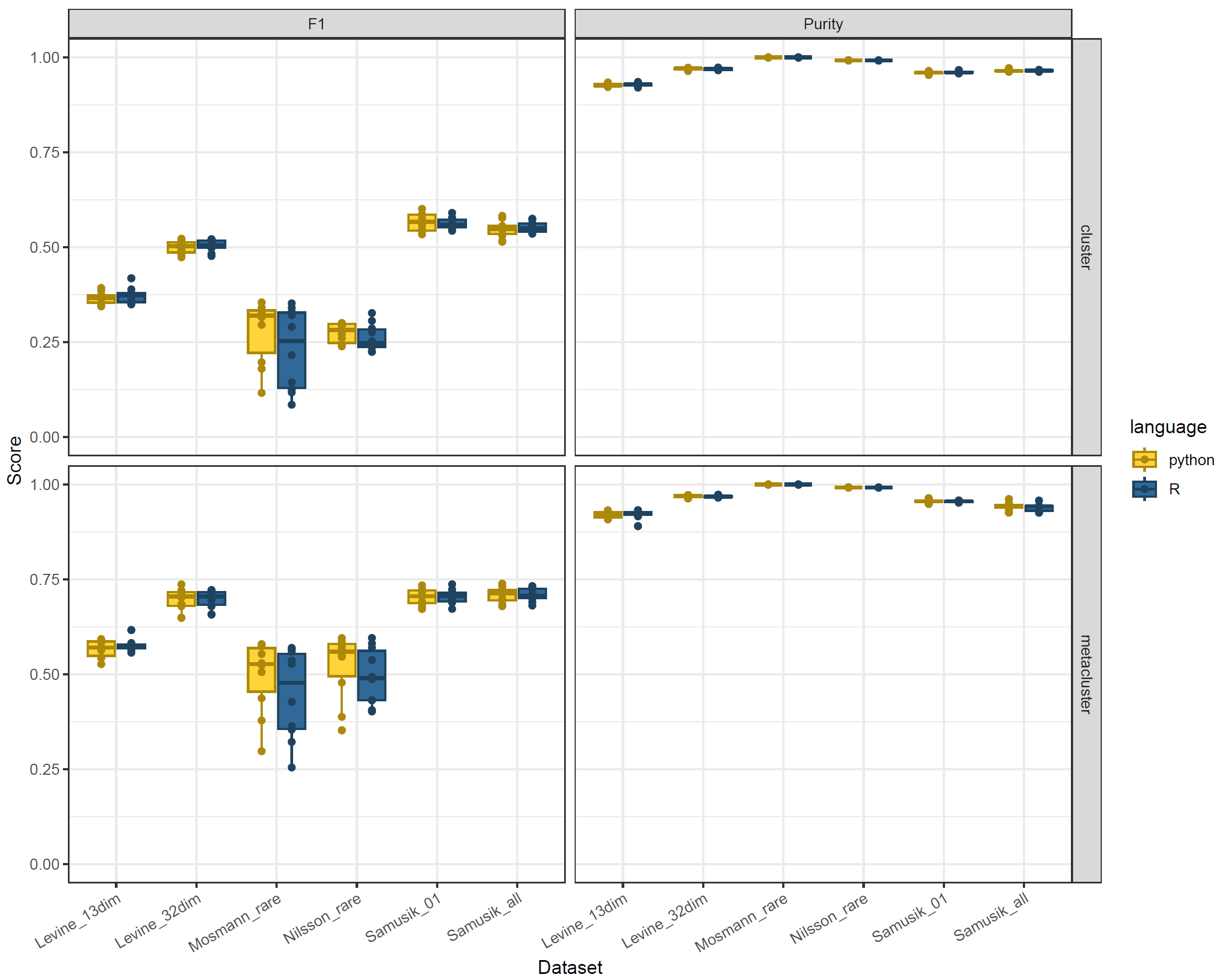
Figure S2: Boxplots show that the performance of the Python implementation of FlowSOM is equal to R.** F1 score and purity are calculated over 10 runs and are based on the manually assigned cell labels and the values predicted from FlowSOM in both Python and R.
